# Assessing Compliance with Evolving Exposure Standards: Respirable Crystalline Silica (RCS) Exposure in Western Australian Mining

**DOI:** 10.3390/ijerph22101567

**Published:** 2025-10-15

**Authors:** Adelle Liebenberg, Kiam Padamsey, Kerry Staples, Matthew Oosthuizen, Marcus Cattani, Andy McCarthy, Jacques Oosthuizen

**Affiliations:** 1 School of Medical and Health Sciences, Edith Cowan University, Joondalup 6028, Australia; a.liebenberg@ecu.edu.au (A.L.); k.padamsey@ecu.edu.au (K.P.); m.oosthuizen@ecu.edu.au (M.O.); m.cattani@ecu.edu.au (M.C.); andymc1178@gmail.com (A.M.); 2 Department of Health (Western Australia), Epidemiology Directorate, Perth 6004, Australia; kerry.staples2@health.wa.gov.au

**Keywords:** respirable crystalline silica, workplace exposure standards, lung disease, mining

## Abstract

The link between occupational exposure to Respirable Crystalline Silica (RCS) and silicosis, a potentially fatal respiratory disease, has been well-established, leading to global reductions in RCS Exposure Standards (ES). In Western Australia (WA), RCS data have been collected by the Department of Energy, Mining, Industry Regulation and Safety (DEMIRS) from 1986 to 2024 (*n* = 144,141). These results were analysed to assess the impacts of recent changes to the ES on compliance. Findings suggest that the WA mining sector, regardless of commodity type, is compliant with RCS exposures as assessed against the 0.05 mg/m^3^ ES (2019). Laboratory technicians, exploratory drilling, miscellaneous trades/utilities, trades assistant, sample preparation, and sampler/sample operator are SEGS that had the highest RCS exposures. Exposure assessment did not account for the protection provided by respiratory protective equipment (RPE). In the WA mining sector, a robust respiratory protection regime is enforced that includes respirator fit testing, and this is most likely the case throughout Australia. On the balance of epidemiological evidence, industry compliance over decades, reducing exposure profiles, and robust RPE programmes, it could be argued that further reductions to the RCS exposure standard are not justified. Regulators need to consider the protection provided by respirators in exposure assessment.

## 1. Introduction

Occupational lung disease remains a significant issue in Australian workplaces. While the incidence of some conditions, such as work-related asthma and asbestosis, appears to be declining, others, such as coal workers’ pneumoconiosis (CWP) and silicosis, are resurging. A national review of occupational lung diseases highlighted major surveillance challenges, citing fragmented data systems and an overreliance on compensation claims that underestimate the true burden of disease [1]. The report called for longitudinal, exposure-rich datasets to improve compliance determinations compared to workplace exposure standards and to monitor emerging risks, particularly for respirable crystalline silica (RCS).

The health effects associated with excessive exposure to RCS are well documented. Classified as a Group 1 carcinogen, the substance is commonly encountered in industries that process materials such as sand, stone, and concrete across sectors, including construction, manufacturing, and mining [2]. Mechanical disruption of these materials through cutting, grinding, or drilling releases microscopic silica particles into the air, posing serious respiratory risks if inhaled. Prolonged inhalation of RCS is an established risk factor for silicosis, a chronic and incurable lung disease marked by inflammation, fibrosis, and progressive loss of lung function [3,4]. Once established, silicosis continues to impair lung function even in the absence of ongoing exposure, significantly diminishing quality of life for workers affected [3]. Effective regulatory standards, management and control strategies, combined with early and accurate diagnosis, including occupational history, radiological evidence, and the exclusion of other respiratory conditions, remain essential in mitigating the progression of the disease [3]. Despite significant advancements in exposure control technologies, recent epidemiological studies continue to demonstrate health risks associated with prolonged low-level chronic exposure to RCS, including increased incidence of silicosis and lung cancer [5]. The resurgence of silicosis and accelerated silicosis from exposure to RCS in the construction industry, particularly for stone benchtop workers, has catapulted RCS exposures to the highest priority and resulted in the banning of silica-containing materials across Australia. There is a difference, though, between exposures in an industry such as construction, where high concentrations are possible over a very short time, and mining, where the silica occurs naturally in rock. These findings reinforce global consensus on the importance of exposure monitoring, improved exposure assessment, and strict enforcement of exposure standards [6].

Despite longstanding recognition of silicosis in mining, global reviews continue to highlight data limitations that hinder effective surveillance [7]. In Australia, a recent surge in accelerated silicosis cases among stonemasons and renewed reports of CWP in the black coal sector prompted a national review of hazardous substances. This review identified RCS as one of nine contaminants requiring enhanced regulatory oversight. In response, updated Workplace Health and Safety (WHS) regulations came into effect nationwide on 1 September 2024, mandating comprehensive management of materials containing ≥1% crystalline silica by weight [8]. These regulations require identification of high-risk tasks likely to generate airborne silica concentrations exceeding the proposed time-weighted average (TWA) exposure limit of 0.025 mg/m^3^, alongside robust exposure monitoring and health surveillance for at-risk workers.

To address the surveillance gaps identified by Alif et al. [1], this study leverages the Western Australian Safety Regulation System (SRS) database, managed by the Department of Local Government, Industry Regulation and Safety (LGIRS), previously the Department of Mines, Energy and Industry Regulation Safety (DEMIRS). This unique dataset, comprising over 50 years of exposure records, enables a longitudinal analysis of RCS exposure trends in the WA mining industry. Specifically, our analysis examined:

1. Trends in exposure: whether RCS exposures in the WA mining industry have decreased over time.
2. Compliance with standards and the extent to which current exposures align with both existing and proposed exposure standards (workplace exposure standards and limits), and
3. SEG-specific risks: whether certain similar exposure groups (SEGs) remain disproportionately exposed.

## 2. Materials and Methods

All data related to mandatory RCS sampling obtained through compliance testing between 1986 and 2024 were extracted by officers employed by DEMIRS from the SRS database Perth, Western Australia, and were provided to the research team as a de-identified MS Excel spreadsheet. Data were analysed using the statistical software R 4.1.0. [9]. Data included in the electronic database included occupation codes, location/site codes, and similar exposure group (SEG) identifiers with corresponding results for respirable crystalline silica (RCS). The data were securely stored in accordance with the Edith Cowan University (ECU), Perth (Joondalup), Western Australia, Data Management plan linked to the project 2023-04914-Oosthuizen that was approved by the human research ethics committee as an out-of-scope project.

Exposure monitoring results contained within the SRS database included compliance testing (mandatory) sampling results. Personal sampling for respirable crystalline silica (RCS) was conducted in accordance with the applicable Australian Standard (AS 2985—Workplace atmospheres method for sampling and gravimetric determination of respirable dust standard), using personal sampling pumps calibrated to a flow rate of 1.9–2.2 L/min with cyclone sampling heads. Sampling is required for a minimum of 75% of the work shift, and samples were analysed by accredited laboratories, and shift adjustments were applied using the Western Australian 12 h shift adjustment model (ACGIH-modified). Results were entered into the SRS database by trained and DEMRIS-approved professionals.

Twenty-three (23) data points with values ≥10 mg/m^3^ were excluded from the analysis in accordance with the approach of An independent analysis of the same regulatory dataset [10] determined these values to likely reflect data entry or measurement error rather than plausible occupational exposures. Summary statistics were produced by commodity type and location (either underground or surface mining operations). The results were compared to historical, current, and proposed ES. The workplace exposure standard (WES) for respirable crystalline silica (RCS) in Australia has decreased from 0.1 mg/m^3^ (1986–2020) to the current 0.05 mg/m^3^ (since 1 July 2020), with regulatory adjustments applied using the Western Australian 12 h shift adjustment model for extended work shifts. Similar Exposure Groups (SEGs) were determined using occupation codes entered in the SRS database. Descriptive and predictive statistics were generated, and mean, 95% lower confidence levels (LCL 95%), and upper confidence levels (UCL 95%) were evaluated to assess compliance for various SEGs. All data was shift-adjusted to account for work rosters common to the WA mining sector, generally 12 h shifts for multiple consecutive days.

### Limitations

In the SRS database, there is a single agent code (SIL) for RCS, and it does not specify whether it is quartz or cristobalite.

## 3. Results

There were 144,141 RCS samples contained within the dataset. To account for extended work schedules typical of the WA mining industry (e.g., 12 h shifts over consecutive days), this dataset was shift-adjusted to standardise exposure durations across samples. Twenty-three samples of 10 mg/m^3^ or more were considered outliers and removed from the analysis, as was performed in the study by Harman et al. [10].

### 3.1. Compliance Trends over Time

**Figure 1:**
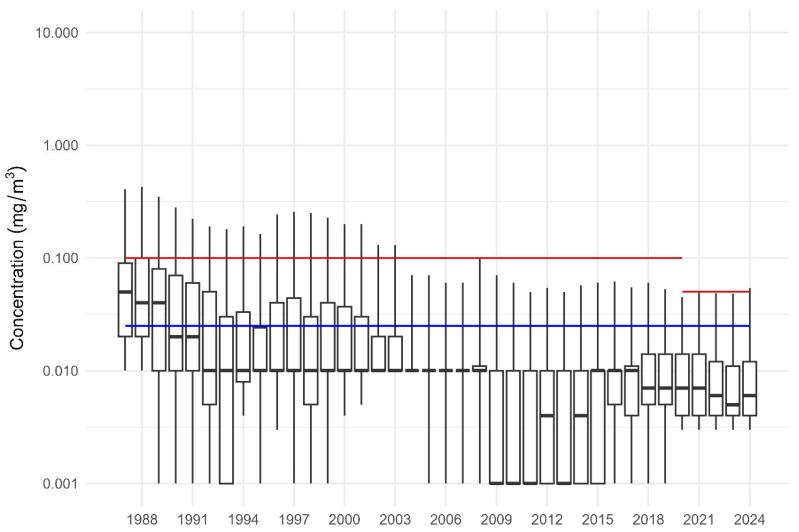
Median RCS concentration with 5th, 25th, 75th and 95th percentiles (1987–2024). Note: The red line from 1986 to 2019 is the repealed ES. The shorter red line from 2019 to 2024 is the current ES, and the blue line represents the proposed ES.

To determine whether RCS exposures in the WA mining industry have decreased over time, and whether current exposure levels are generally compliant with the existing ES, the overall mean and 95% upper confidence level (UCL95%) for RCS were calculated as depicted in Figure 1. Over this period, RCS exposures decreased, with the mean and UCL95% remaining consistently below both the 0.1 mg/m^3^ (pre-2019 the red line) and 0.05 mg/m^3^ (post-2019the short red line) ES. Sample means have remained consistent from 2009 onwards, and based on 95% CI and mean values, there were no exceedances of the ES from 2020 to 2024; in fact, the industry has been compliant with the proposed ES of 0.05 mg/m^3^ (blue line).

### 3.2. Differences Between Occupational Groups

To determine if specific occupational groups (SEGS) had more exceedances of the ES than others, SEGs with at least ten samples, and with both a high GSD and UCL above 0.05 mg/m^3^ (post-2019 ES) were identified for 2024, and for the period from 2020 to 2024 combined. These descriptive statistics (mean, minimum and maximum values, standard deviation (SD), lower and upper confidence limits (LCL, UCL), and geometric standard deviation (GSD)) provide insight into the variability and skewness of the data, which generally shows a downward trend in exposures over time.

**Figure 2:**
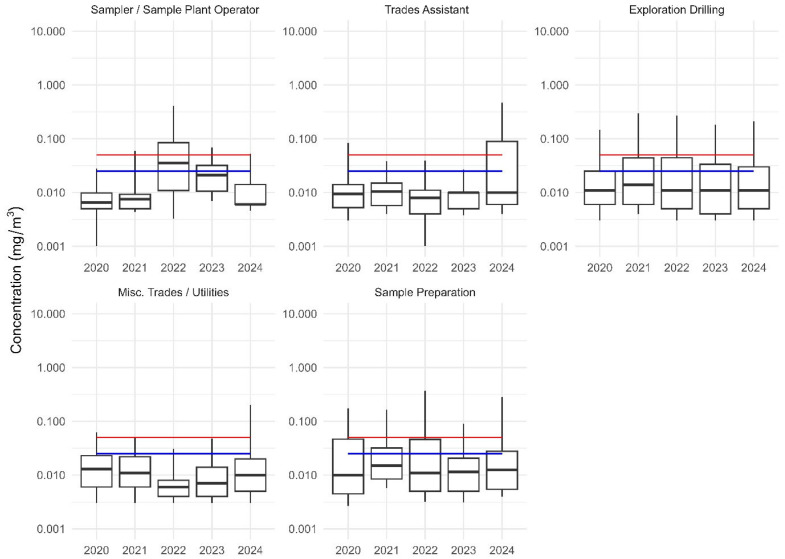
Median RCS concentration for selected SEGs with 5th, 25th, 75th and 95th percentiles (2020 to 2024). The red lines are the repealed ES. The blue lines represents the proposed ES.

Over the last 5 years, the most exposed workers were those employed in the exploratory drilling, miscellaneous trades/utilities, trades assistant, sample preparation, sampler/sample plant SEGS. The annual mean and 95% CI are shown in Figure 2.

## 4. Discussion

This study aimed to evaluate RCS exposure trends within the WA mining industry, focusing on three core objectives. First, it investigated whether RCS exposures have declined over time, reflecting the effectiveness of industry-wide monitoring and control measures. Second, it assessed the extent to which current and proposed ES are being met across various mining operations. Finally, SEG-specific exposures were analysed to determine whether certain similar SEGs remain disproportionately exposed, despite overall reductions in industry-wide exposure levels.

### 4.1. Trends over Time

The results of this study indicate a significant reduction in RCS exposures in the WA mining industry between 1986 and 2024. Stratified analysis by commodity between 2014 and 2024 revealed a general downward trend in exposure levels, although intermittent peaks were observed in specific years and commodities. These fluctuations lacked a consistent explanatory pattern but remained below applicable ES thresholds.

This trend aligns with findings from Harman et al. [10], highlighting variability in exposure levels, with wide confidence intervals and high geometric standard deviations (GSDs), particularly in exploration drilling and laboratory roles. Together, these findings suggest that industry-wide investment in exposure control technologies and monitoring within the State of WA has been effective in reducing average exposures over time.

### 4.2. Compliance with Exposure Standards

Western Australia proactively regulates and enforces comprehensive management strategies for RCS exposure, and the state regulatory authority for mines (LGIRS) utilises the SRS, a purpose-built, online reporting and monitoring platform, to systematically track and manage exceedances of exposure limits. Available data suggest that the mining industry in WA has consistently met regulatory obligations for RCS exposures, with most of the reported mean measurements for RCS since the 1990s below the ES relevant at the time of collection. This is confirmed by an independent study conducted in 2023 [11]. While compliance with the current ES offers a high level of protection, differences in roster patterns and exposure durations highlight the need to consider cumulative exposure in long-term risk assessments.

In 2019, the ES for RCS was halved from 1 mg/m^3^ to 0.05 mg/m^3^, and it is likely to be reduced even further to 0.025 mg/m^3^ [12]. Industry should proactively strive to achieve exposure levels lower than 0.05 mg/m^3^. Continued improvements in exposure monitoring and sampling methodologies will not only ensure compliance but may also enhance worker safety by reducing the cumulative health risks associated with chronic RCS exposure.

The ES provides a regulatory threshold that is suitable for daily exposure; however, another useful metric for evaluating long-term health risk, particularly in dose–response studies of diseases such as silicosis, is cumulative exposure (expressed as mg/m^3^-years). A recent systematic review and dose–response meta-analysis [13] demonstrated that reducing cumulative RCS exposure from 4 mg/m^3^-years (equivalent to 40 years at 0.1 mg/m^3^) to 2 mg/m^3^-years (40 years at 0.05 mg/m^3^) was associated with a substantial reduction in silicosis risk. This highlights the importance of maintaining low daily exposures over time. Applying the findings of Howlett et al. [13], it is determined that RCS exposure at the current ES, for workers on an 8 h/day, 5 days a week roster (averaging 40 h per week), would result in a cumulative exposure of approximately 2 mg/m^3^ over 40 years. However, for those on a 12 h shift, two weeks on, one week off roster (averaging 56 h per week), the same daily exposure concentration results in faster cumulative doses, reaching 2 mg/m^3^ in under 30 years, thus providing further support for reductions in RCS exposure levels.

While our analysis was restricted to describing shift-adjusted daily exposure profiles, the implications of cumulative exposure over months or years have been explored in detail by Harman et al. [10] using the same dataset. Their modelling of these data provides important complementary insights into the long-term health risks associated with RCS exposure. In WA, exposure data and silicosis incidence have been recorded since 1925, and up until the 1970s, there were between 50 and 100 cases reported annually [10]. During the period from 1925 to 1996, mine workers were required to have regular chest X-rays, and in 1974, the ES for RCS was set at 0.2 mg/m^3^. This resulted in a significant reduction in silicosis incidence, and personal breathing zone monitoring was introduced in 1986 [11]. Other independent studies in 1993 [11], 2002 [14], and 2017 [15] found no silicosis cases in WA amongst those who entered the workforce after 1974. The ES was subsequently reduced further to 0.1 mg/m^3^ in 2005 and 0.05 mg/m^3^ in 2020 [12]. Since 2021, LGIRS has mandated health surveillance that includes a medical examination, spirometry, and low-dose high-resolution computed tomography (LDCT) screening for silicosis, and this data is captured by the Safety Regulatory System (SRS), which is the health and exposure database of LGIRS [10]. Harman et al. [10] analysed the SRS dataset (up to 2023) to identify mining worker groups that may be at an elevated risk of developing RCS-associated lung pathology by linking personal exposure results with disease surveillance data. They reported that exposure levels have been stable over the last 2 decades; however, exploratory drillers and laboratory workers have relatively higher exposures.

One of the significant issues that needs to be considered by any exposure monitoring is the influence of respiratory protection, since all sampling is conducted in the breathing zone but outside of respirators. This will change in Australia from December 2026, when the industry will be allowed to consider the protection provided by RPE [12]. In the WA mining sector, a robust respiratory protection regime is enforced that includes RPE fit testing [10], and this is most likely the case throughout Australia. On the balance of epidemiological evidence, industry compliance over decades, reducing exposure profiles, and robust RPE programmes, it could be argued that further reductions to the RCS exposure standard are not justified.

### 4.3. SEG-Specific

While overall compliance has been achieved, SEG-specific analysis reveals persistent variability in exposure levels. The highest RCS exposure levels were found in base metal mining and exploration settings, with drilling occupations among the most highly exposed jobs. Harman et al. [10] revealed that occupations such as exploration drilling assistants, trades assistants, sample preparation workers, and laboratory technicians consistently show higher exposure levels and greater variability, as indicated by elevated GSDs and wide confidence intervals, and suggested elevated long-term health risks for these cohorts. This finding is supported in our analysis.

In high-risk SEGs such as these, the routine use of RPE should be considered standard practice. This must be supported by comprehensive RPE management programmes that include respirator fit testing, training, and enforcement of clean-shaven policies to ensure effective protection. Furthermore, there is a need to establish a standardised correction factor for samples collected in the breathing zone outside of the RPE. These should incorporate an allowance for the uncertainty associated with respirator fit and user compliance. Addressing these methodological challenges will be essential to ensure that exposure assessments remain both accurate and protective as regulatory thresholds become more stringent [12].

As workplace exposure standards continue to decrease, occupational hygienists will need to adapt sampling and reporting methodologies accordingly. Regulatory frameworks must also evolve to allow for the accurate reporting of worker exposures that account for the protection factors provided by RPE. This includes the development of validated methods for placing samplers inside the respirator to verify actual exposure levels, rather than relying solely on theoretical protection estimates.

## 5. Conclusions

The WA mining sector is deemed to be generally compliant when mean RCS exposures are assessed against the 2019 ES of 0.05 mg/m^3^. Although overall exposures are compliant, some SEGs, such as exploration drilling, laboratory technicians, and sample preparation personnel, recorded relatively higher exposure levels, and such groups require more frequent monitoring and mandated use of RPE as a precautionary measure. Wearing respiratory protective equipment (RPE) in at-risk SEGs is already industry norm and includes well-developed RPE management programmes with respirator fit testing and clean-shaven policies. Regulators need to develop procedures that will enable organisations to report worker exposures while accounting for RPE protection factors. Furthermore, the regulator needs to develop processes to detail how compliance with requirements for approved RPE programmes will be regulated and the development of methods to apply standardised correction factors to samples collected in the breathing zone, external to the RPE.

## Data Availability

All data supporting the findings of this study are contained within the confidential DEMIRS database of the Western Australian Government. The extracted de-identified data can be requested from the corresponding author.
